# Phase II Clinical Trial With Pegylated Liposomal Doxorubicin (CAELYX®/Doxil®) and Quality of Life Evaluation (EORTC QLQ-C30) in Adult Patients With Advanced Soft Tissue Sarcomas: A study of the Spanish Group for Research in Sarcomas (GEIS)

**DOI:** 10.1080/13577140500287024

**Published:** 2005

**Authors:** A. Poveda, A. López-Pousa, J. Martín, J. García Del Muro, R. Bernabé, A. Casado, C. Balañá, O. Sanmartín, M. D. Menéndez, P. Escudero, J. Cruz, Elena Belyakova, D. Menéndez, J. M. Buesa

**Affiliations:** 1 Instituto Valenciano de Oncología Valencia Spain; 2 Hospital de San Pau Barcelona Spain; 3 Hospital Son Dureta Palma de Mallorca Spain; 4 Instituto Catalán de Oncología Barcelona Spain; 5 Hospital Virgen del Rocío Sevilla Spain; 6 Hospital San Carlos Madrid Spain; 7 Hospital Germán Trías y Pujol Badalona Spain; 8 Hospital Provincial de Conxo Santiago de Compostela Spain; 9 Hospital Clínico Zaragoza Spain; 10 Hospital Universitario de Canarias La Laguna Spain; 11 Biostatician Instituto Universitario de Oncología del Principado de Asturias (IUOPA) Spain; 12 Data Manager Hospital de San Pau Barcelona Spain; 13 Servicio de Oncología Médica Hospital Central de Asturias (IUOPA) Julián Clavería s/n Oviedo 33005 Spain

## Abstract

*Background:* Pegylated liposomal doxorubicin (PLD), a formulation with pharmacokinetic differences with respect to doxorubicin (DXR), might benefit patients with advanced soft tissue sarcoma (STS) pretreated with DXR.

*Patients and methods:* Patients with measurable and progressive STS received PLD at 35 mg/^2^ every 3 weeks. Quality of life before and during treatment was assessed with EORTC QLQ-C30.

*Results:* Twenty-eight patients, 22 DXR-pretreated, were given 140 cycles (median 3, range 1–18). Activity in 27 patients (5 GIST): one complete and one partial remission (both non-GIST and without prior DXR), 12 stabilizations and 13 progressions (response rate 7.4%, 95% CI: 0–17%). Grade 3 toxicity: palmar-plantar erythrodysesthesia (19% of patients), stomatitis (4%) or cutaneous (4%). Neutropenia grade≥3 was detected in 16% of patients. Median relative dose intensity was 95%. Progression-free rate at 3 and 6 months was, respectively, 48 and 22%, median progression-free survival 5.8 months and median overall survival 8.7 months. QLQ-C30 at baseline and at weeks 6–11 in 23 and 13 patients, respectively, showed good reliability and validity. Quality of life did not seem to worsen during therapy.

*Conclusions:* PLD did not induce objective remissions in 22 STS patients pretreated with DXR, but progression-free rate figures support the use of this agent in patients who have not progressed under a DXR-containing regimen. The toxicity observed was comparable to that of other PLD schedules.


*Sarcoma*, September/December 2005; 9(3/4): 127–132

Taylor & Francis

Taylor & Francis Group

ORIGINAL ARTICLE

### Phase II clinical trial with pegylated liposomal doxorubicin (CAELYX®/Doxil®) and quality of life evaluation (EORTC QLQ-C30) in adult patients with advanced soft tissue sarcomas

### A study of the Spanish Group for Research in Sarcomas (GEIS)

A. POVEDA[1], A. LÓPEZ-POUSA[2], J. MARTÍN[3], J. GARCÍA DEL MURO[4], R. BERNABÉ[5], A. CASADO[6], C. BALAÑÁ[7], O. SANMARTÍN[1], M. D. MENÉNDEZ[8], P. ESCUDERO[9], J. CRUZ[10], ELENA BELYAKOVA[11], D. MENÉNDEZ[12], & J. M. BUESA[13]

[1]*Instituto Valenciano de Oncología, Valencia,* [2]*Hospital de San Pau, Barcelona,* [3]*Hospital Son Dureta, Palma de Mallorca,* [4]*Instituto Catalán de Oncología, Barcelona,* [5]*Hospital Virgen del Rocío, Sevilla,* [6]*Hospital San Carlos, Madrid,* [7]*Hospital Germán Trías y Pujol, Badalona,* [8]*Hospital Provincial de Conxo, Santiago de Compostela,* [9]*Hospital Clínico, Zaragoza,* [10]*Hospital Universitario de Canarias, La Laguna,* [11]*Biostatician, Instituto Universitario de Oncología del Principado de Asturias (IUOPA),* [12]*Data Manager, Hospital de San Pau, Barcelona, and* [13]*Hospital Central de Asturias (IUOPA), Oviedo, Spain*

*(Received 8 April 2005; revised 5 July 2005; accepted 22 July 2005)*

**Abstract**
*Background*: Pegylated liposomal doxorubicin (PLD), a formulation with pharmacokinetic differences with respect to doxorubicin (DXR), might benefit patients with advanced soft tissue sarcoma (STS) pretreated with DXR.
*Patients and methods*: Patients with measurable and progressive STS received PLD at 35 mg/m$^2$ every 3 weeks. Quality of life before and during treatment was assessed with EORTC QLQ-C30.
*Results*: Twenty-eight patients, 22 DXR-pretreated, were given 140 cycles (median 3, range 1–18). Activity in 27 patients (5 GIST): one complete and one partial remission (both non-GIST and without prior DXR), 12 stabilizations and 13 progressions (response rate 7.4%, 95% CI: 0–17%). Grade 3 toxicity: palmar-plantar erythrodysesthesia (19% of patients), stomatitis (4%) or cutaneous (4%). Neutropenia grade $\geq$3 was detected in 16% of patients. Median relative dose intensity was 95%. Progression-free rate at 3 and 6 months was, respectively, 48 and 22%, median progression-free survival 5.8 months and median overall survival 8.7 months. QLQ-C30 at baseline and at weeks 6–11 in 23 and 13 patients, respectively, showed good reliability and validity. Quality of life did not seem to worsen during therapy.
*Conclusions*: PLD did not induce objective remissions in 22 STS patients pretreated with DXR, but progression-free rate figures support the use of this agent in patients who have not progressed under a DXR-containing regimen. The toxicity observed was comparable to that of other PLD schedules.

**Keywords:** *Pegylated liposomal doxorubicin, soft tissue sarcomas, quality of life*

### Introduction

For advanced soft tissue sarcomas (STS) of the adult, doxorubicin is one of the drugs which has shown a 20–30% objective activity [1], but total dose is limited by the risk of developing myocardiopathy, a side effect related to DXR peak plasma concentrations as well as cumulative dose. Liposomal technology has been applied to achieve a slower DXR liberation, in an attempt to overcome this problem. In pegylated liposomal doxorubicin (PLD), polyethylene glycol provides an outer layer that protects the liposome from uptake by the reticuloendothelial system. This facilitates the distribution of liposomes to the tissues and to the tumor bed, and DXR plasma half-life is considerably

Correspondence: José M. Buesa, Servicio de Oncología Médica, Hospital Central de Asturias, Julián Clavería s/n, 33005 Oviedo, Spain. Tel: 34 985 106121. Fax: 34 985 234498. E-mail: jmbuesa@hca.es, jmbuesap@seom.org

ISSN 1357–714X print/ISSN 1369–1643 online/05/(03-04)0127-6 © 2005 Taylor & Francis
DOI: 10.1080/13577140500287024

prolonged, total body clearance being reduced 250-fold when compared to equivalent DXR doses, what may influence its antitumor efficacy [2,3]. This pharmacokinetic behavior influences the toxicity profile of PLD, so that palmar-plantar erythrodysesthesia (PPE) or mucositis are usually dose-limiting, hematological toxicity is less accentuated, and cardiotoxicity is lower [3–5]. PLD toxicity is schedule-dependent, and can be modified by reducing the dose or by increasing the dose interval [4,6,7].

In 1997 our Group planned a Phase II study with PLD in STS patients to determine whether this DXR formulation could provide some benefit to patients already exposed to chemotherapy, including DXR. Recommended doses varied from 50 to 60 mg/m$^2$ every 4–6 weeks [6–8] and, in an attempt to reduce toxicity, we selected a dose of 35 mg/m$^2$ repeated every 3 weeks, which offered a dose-intensity similar to that of previously tested schedules. Another objective of the trial was to assess the quality of life of patients during PLD therapy.

### Patients and methods

### *Patients*

Patients should have a histological STS diagnosis; advanced, measurable and unresectable progressive disease after DXR and ifosfamide; performance status $\leq 2$ (WHO); adequate bone marrow (haemoglobin > 9 g/dl, granulocytes $> 1.5 \times 10^9$/l and platelets $\geq 100 \times 10^9$/l), liver (serum bilirubin, AST and ALT values < 2 UNL) and renal (serum creatinine < 1.5 UNL) functions; left ventricular ejection fraction (LVEF) $\geq$50%; prior cumulative dose of doxorubicin $\leq$300 mg/m$^2$. Patients with NYHA class $\geq$II cardiomyopathy, or radiotherapy over the only measurable lesion, were excluded. Patients progressing $\leq$6 months from any DXR-containing regimen for advanced disease, or <12 months if adjuvant, were considered resistant to DXR. Ethics Committee of participating institutions approved the study and informed patients signed a consent form.

### *Treatment and evaluation criteria*

The drug for this study was kindly provided by Schering-Plough España S.A. All patients received PLD (Caelyx®, Doxil®) at 35 mg/m$^2$ over 1 h, every 3 weeks.

Toxicity was evaluated according to NCI Common Toxicity Criteria, version 1.0. Cycles were repeated on day 21 if granulocytes $>1.0 \times 10^9$/l and any other toxicity had reached grade $\leq 1$. If granulocyte nadir was $<0.5 \times 10^9$/l, the dose was reduced by 25%. Patients with grade 1 PPE, stomatitis, esophagitis or cutaneous toxicity on day 21, who had not suffered a similar episode of grade 3–4, repeated the cycle on schedule; otherwise, cycle was delayed until complete recovery, and the dose reduced by 25% if a 2-week delay was necessary. Patients were removed from study if grade 3–4 toxicity was still present at day 35 or if >2 dose reductions were necessary. Patients with a confirmed LVEF < 45% abandoned the study.

Patients were controlled weekly for toxicity. LVEF was measured every 6 weeks. Only cycles with weekly BCC were analyzed for hematological toxicity, and patients receiving one PLD dose were assessed for toxicity. Target lesions were evaluated every two cycles (or 6 weeks). WHO criteria for activity were applied [9] and patients continued on study until disease progression or excessive toxicity occurred. Time to progression, progression-free survival and overall survival were estimated by actuarial methods.

Authorization to utilize the quality of life questionnaire C30 (QLQ-C30), version 1.0 (10), was obtained from the EORTC Quality of Life Unit. It was to be administered to patients before treatment and every other cycle. Scoring was performed according to the EORTC QLQ-C30 Scoring Manual recommendations [11].

### *Statistics*

Sample size was calculated according to a minimax-design [12]. For $P_0 = 10\%$ and $P_1 = 25\%$ DXR activity in first line, and $\alpha$ and $\beta$ errors equal to 0.1, the initial sample size was 27 patients. If two or less responses were observed in the first 27 patients, the study would be considered not worth expanding.

Evaluation of QLQ-C30 results were performed as recommended [10]. The internal consistency of QLQ-C30 was assessed by Cronbach's $\alpha$ coefficient, with values >0.70 considered acceptable [13]. Pearson's $r$ was calculated to check the validity of the questionnaire, and values >0.40 indicated substantial correlations among the different scales.

### Results

### *Patient characteristics*

From June 1998 to June 1999, 28 patients entered this study (Table I). One of the six patients with a retrospective GIST diagnosis died of tumor-related complications 2 weeks after the first cycle and was not evaluable. Another patient received only one cycle due to rapid disease progression. In total, 140 PLD cycles were delivered, (median 3, range 1–18). All patients had received one prior chemotherapy regimen (24 for advanced disease, four adjuvant) that in 23 had included DXR. Median cumulative dose of doxorubicin per patient was 200 mg/m$^2$ (range 50–300). Eleven out of 18

Table I. Patient characteristics.

| | |
|---|---|
| *Number* | 28 |
| Male/Female | 15/13 |
| Age (median, range) | 54.4 (28–74) |
| *Performance status* | |
| 0 | 10 |
| 1 | 14 |
| 2 | 4 |
| *Histological type of sarcoma* | |
| GIST | 6 |
| Leiomyosarcoma | 4 |
| Malignant fibrous histiocytoma | 3 |
| Synovial sarcoma | 3 |
| Liposarcoma | 2 |
| Neurogenic sarcoma | 2 |
| Other | 8 |
| *Grade of malignancy* | |
| 1 | 2 |
| 2 | 10 |
| 3 | 16 |
| *Primary site* | |
| Extremities and trunk wall | 18 |
| Gastrointestinal | 5 |
| Uterine | 3 |
| Other | 2 |
| *Sites of disease* | |
| Lung | 19 |
| Primary tumor | 6 |
| Liver | 6 |
| Intra-abdominal | 4 |
| Lymph node | 5 |
| Soft-tissues | 2 |
| *Prior chemotherapy* | |
| Doxorubicin ± ifosfamide | 23 |
| High-dose ifosfamide | 3 |
| Ifosfamide ± cis-platinum | 2 |
| Treatment-free interval (months) (median, range) | 4.6 (0.83–37) |

non-GIST patients were resistant and seven were potentially sensitive to DXR, with all GIST patients being resistant.

### *Toxicity*

Maximum hematological toxicity noted was anemia of grade 3 in 4% of patients, granulocytopenia grade 3–4 in 16% and thrombocytopenia grade 2 in 4%. Toxicity on granulocytes was not cumulative and nadir occurred by day 21 (median). PPE reached grade 3 in five patients, three of whom had to abandon the study for this toxicity (Table II). PPE grade 3 was noted after a median of five cycles (range 3–8), with a cumulative incidence in cycles 3–8 of 1.4, 1.1, 4, 4.6, 5 and 6%, respectively. A patient with persistent grade 2 stomatitis in spite of two dose reductions was also removed from the study. Cutaneous toxicity was described as a psoriasiform eruption in one patient, and as a pruriginous erythema in 10, only in one reaching grade 3 concomitantly with grade 3 PPE. In this patient the lesions had a lichenoid appearance and a diagnosis of neoplastic epidermal toxicity was made upon biopsy [14].

Table II. Maximum grade of toxicity observed per patient.

| Grade | 0 | 1 | 2 | 3 |
|---|---|---|---|---|
| Nausea | 61 | 34 | 4 | – |
| Vomiting | 73 | 19 | 8 | – |
| Stomatitis | 42 | 23 | 31 | 4 |
| Esophagitis | 85 | 15 | – | – |
| Cardiac | 92 | 8 | – | – |
| Cephalea | 84 | 16 | – | – |
| Asthenia | 39 | 31 | 19 | 11 |
| Anorexia | 58 | 19 | 15 | 8 |
| Fever | 73 | 27 | – | – |
| Infection | 85 | 11 | – | 4 |
| Pruritus | 85 | 11 | 4 | – |
| Cutaneous | 62 | 15 | 19 | 4 |
| PPE | 34 | 34 | 12 | 19 |
| Alopecia | 90 | 5 | 5 | – |

Figures represent percentage of patients.

Asthenia of grade 3 was noted in three patients and infection of grade 3, unrelated to therapy, occurred in one patient. No hypersensitivy reactions or episodes of cardiac failure were registered. Median cumulative anthracycline (prior doxorubicin plus PLD) was 350 mg/m$^2$ (range 70–577). Two out of 17 patients with serial LFEV showed a transient decrease of 25 and 14%, respectively. PLD was delayed a median of 7 days (range 7–14) in 21 cycles, due to hematological (6), muco-cutaneous toxicity (13) or other reasons (2), and it was reduced by 25% in five patients. Cumulative PLD was 141 mg/m$^2$ (35–567), dose intensity 11 mg/m$^2$/week (7–12), and relative dose intensity of 95% (0.61–1.03) (median, range).

### *Response to therapy and survival*

Objective activity in 27 evaluable patients included one complete (CR) and one partial remission (PR), 12 stabilizations (NC) and 13 progressions (PD) (response rate 7.4%; 95% CI, 0–17%). One out of five patients with a GIST diagnosis had a <50% reduction of liver metastases, one had disease stabilization and three progressed. If GIST patients are excluded, the remission rate was 9% (95% CI, 0–21%). Objective remissions were noted in two out of five patients without prior DXR exposure after five and 18 PLD cycles, and lasted 6 months. In 22 patients with prior DXR treatment, 12 stabilizations and 10 progressions were noted. Median duration of stable disease was 4.7 months (range 2.7–12 months). Among non-GIST patients, 11 DXR-resistant had three stabilizations and eight progressions, with seven stabilizations noted in seven patients with potentially DXR-sensitive disease (Fisher exact test, $P = 0.004$). Progression-free rate (PFR) at 3 and 6 months was, respectively, 48 and 22% for the whole group, 40 and 40% for

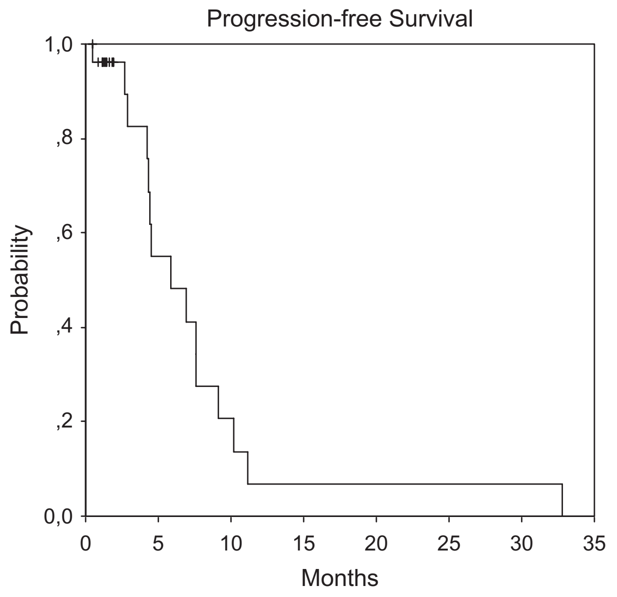

Figure 1. Progression-free survival.

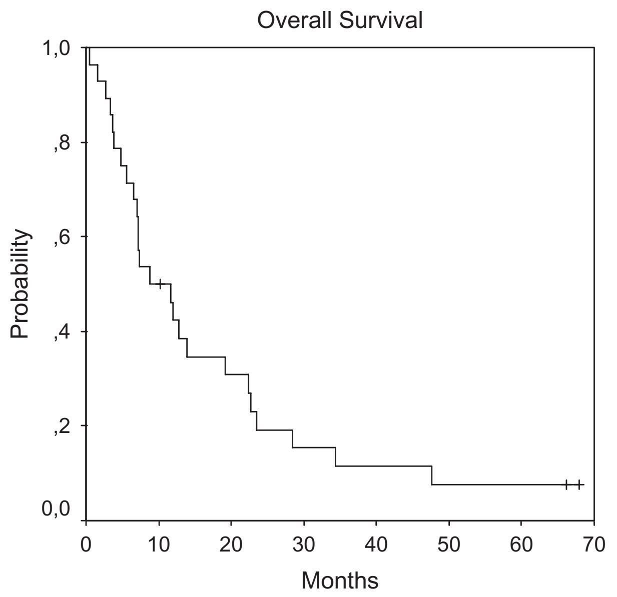

Figure 2. Overall survival.

patients never exposed to DXR, and 45 and 18% for those with prior DXR therapy. Median progression-free survival was 5.8 months (95% CI: 1.4–10 months) (Figure 1), and all but two patients have died with a median overall survival of 8.7 months (95% CI: 2.8–15 months) (Figure 2).

### *Quality of life*

A baseline QLQ-C30 was available from 23 patients, with evaluations obtained from 13 patients at weeks 6–11, and from seven patients at weeks 12–18. In Table III we present mean score values ($\pm$standard deviation) of the different scales with the corresponding Cronbach's $\alpha$ coefficients for multi-item scales. All correlations among QLQ-C30 scales before treatment were significant ($P<0.056$); those same correlations within weeks 6–11 ($n=13$) were significant, except for the pairs global quality of life–physical ($P=0.16$), fatigue–social ($P=0.13$) and nausea and vomiting–role ($P=0.10$) (data not shown). No significant differences were noted when scores at weeks 6–11 or at week >12 were paired-compared with the respective baseline values. Correlation of the initial WHO performance status and the scale for global quality of life (QLQ-C30) at baseline showed a certain trend for lineal correlation ($V_{Cramer}=0.753$ and contingency coefficient $=0.794$, both with $P=0.062$).

### Discussion

The activity of PLD administered at 35 mg/m$^2$ every 3 weeks to patients with STS previously treated with doxorubicin, measured in terms of objective remissions, is negligible. Prior studies have been conducted with PLD doses that varied from 50 to 60 mg/m$^2$ repeated every 3–4 weeks. In two trials, where most patients had received DXR, five out of 45 STS patients responded [15, 16], and two small studies conducted in previously untreated patients gave negative results [17,18]. In a first-line comparative study of the EORTC STBSG, 50 patients received PLD and 45 DXR, with a remission rate, respectively, of 10 and 9%, with no differences detected in time to progression or overall survival [19]. Progression-free rate has been proposed as a parameter to evaluate the activity of new agents in patients with advanced STS. In this trial, progression-free rates at 3 and 6 months were higher than the 39 and 14% encountered, respectively, for active drugs delivered as second-line regimens [20]. Those figures may be explained by a potential DXR sensitivity in our patient population, as 50% of non-GIST patients had either not received DXR or not progressed during a DXR-containing regimen, supporting the administration of PLD to patients who have not progressed under DXR therapy.

Phase II trials conducted with PLD have been usually performed at 50 mg/m$^2$ delivered every 4 weeks, with stomatitis grade 3–4 noted in 4–9% of patients and PPE grade 3–4 in 17–20% [19,21]. In this trial PPE appeared in 65% of patients and stomatitis grade 2–3 was reported by 35%. Intensity and incidence of stomatitis and PPE have been related, respectively, to dose and cycle interval. Grade 2–4 stomatitis occurred in 83% of patients receiving PLD at 70 mg/m$^2$ every 6 weeks, and grade 2–3 PPE appeared in 73% of those treated at

Table III. Score of the EORTC QLQ-C30 before and during treatment.

| | Before treatment (*n* = 23) | | | During treatment (weeks 6–12) (*n* = 13) | | | During treatment (week > 12) (*n* = 7) | | |
|---|---|---|---|---|---|---|---|---|---|
| | Mean score | SD | $\alpha$ | Mean score | SD | $\alpha$ | Mean score | SD | $\alpha$ |
| Functioning scales | | | | | | | | | |
| Physical | 63.5 | 32.8 | 0.82 | 63.8 | 26.9 | 0.72 | 62.8 | 34.02 | 0.81 |
| Role | 54.3 | 45 | 0.74 | 51.9 | 41.4 | 0.50 | 82.1 | 37.40 | 0.93 |
| Cognitive | 84 | 24 | 0.87 | 91 | 12.9 | 0.04 | 85.8 | 17.21 | 0.90 |
| Emotional | 72.4 | 22.2 | 0.80 | 72.4 | 27 | 0.89 | 61.2 | 22.22 | 0.87 |
| Social | 67.4 | 33.5 | 0.80 | 66.6 | 35.3 | 0.91 | 69.2 | 26.95 | 0.79 |
| Global quality of life | 50 | 26.3 | 0.96 | 53.8 | 22.7 | 0.96 | 64.6 | 12.56 | 0.93 |
| Symptom scales and/or items | | | | | | | | | |
| Fatigue | 33.8 | 25.2 | 0.89 | 25.2 | 25.5 | 0.94 | 18.7 | 13.50 | 0.70 |
| Nausea and vomiting | 11.6 | 29 | 0.97 | 11.5 | 20.8 | 1 | 8.3 | 12.73 | 0.75 |
| Pain | 34 | 26.3 | 0.79 | 19.2 | 16.4 | 0.68 | 30.6 | 24.57 | 0.92 |
| Dyspnea | 7.2 | 14 | | 10.2 | 21 | | 9.5 | 25.2 | |
| Sleep disturbance | 27.5 | 31.2 | | 30.7 | 31.8 | | 19 | 17.8 | |
| Appetite loss | 27.5 | 34.3 | | 25.6 | 33.7 | | 28.6 | 30 | |
| Constipation | 18.8 | 30 | | 12.8 | 21.7 | | 19 | 37.80 | |
| Diarrhea | 2.9 | 13.9 | | 5.1 | 12.5 | | 4.7 | 12.6 | |
| Financial impact | 21.7 | 35.6 | | 38.4 | 42.7 | | 38.9 | 44.3 | |

$\alpha$ = Cronbach's alpha coefficient. Values $\geq$ 0.70 indicate adequate scale reliability.

35 mg/m$^2$ every 3 weeks, both schedules with a dose intensity of 11.6 mg/m$^2$ per week, equivalent to ours [22]. Mucositis was also dose-limiting in another trial with 70 mg/m$^2$ PLD delivered every 6 weeks [23]. The schedule we tested did not cause a higher PPE incidence than regimens where PLD was delivered every 4 weeks and, in our case, the intensity of stomatitis was lower. Grade 3–4 granulocytopenia (16% of patients) was higher than the 2–6% reported with PLD at 50 mg/m$^2$ every 4 weeks [19,21], but in our trial 93% of cycles had weekly BCCs. No episodes of cardiac failure were observed, and the lower cardiac toxicity of PLD with respect to DXR has been confirmed in a recent study [24].

Quality of life before treatment was measured in 23 patients with the EORTC QLQ-C30 (version 1.0). To our knowledge, this questionnaire had not been passed before to STS patients, and the limited data obtained here point to its adequacy to measure the quality of life of sarcoma patients exposed to chemotherapy. In 13 patients with assessable data, quality of life did not seem to worsen during therapy.

### Acknowledgements

Supported in part by a grant from Schering-Plough España S.A. This study was completed within the framework of Program 9 of the Redes Temáticas de Investigación Cooperativa de Centros de Cáncer (Grant C03/10), Instituto de Salud Carlos III, Ministerio de Sanidad y Consumo, Spain. The Instituto Universitario de Oncología del Principado de Asturias (IUOPA) is supported by Obra Social Cajastur, Asturias, Spain.

### References

1. Brennan MF, Casper ES, Harrison LB. Soft tissue sarcoma. In DeVita VT, Hellman S, Rosenberg SA, editors. Cancer: Principles and practice of oncology. 5th ed. Philadelphia, PA: Lippincott-Raven, 1997. pp 1738–1853.
2. Coukell AJ, Spencer CM. Polyethylene glycol-liposomal doxorubicin. A review of its pharmacodynamic and pharmacokinetic properties, and therapeutic efficacy in the management of AIDS-related Kaposi's sarcoma. Drugs 1997;53:520–538.
3. Gabizon A, Shmeeda H, Barenholz Y. Pharmacokinetics of pegylated liposomal doxorubicin: Review of animal and human studies. Clin Pharmacokinet 2003;42:419–436.
4. Uziely B, Jeffers S, Isacson R, et al. Liposomal doxorubicin: Antitumor activity and unique toxicities during two complementary phase I studies. J Clin Oncol 1995;13: 1777–1785.
5. Ranson MR, Carmichael J, O'Bryne K, et al. Treatment of advanced breast cancer with sterically-stabilized liposomal doxorubicin: Results of a multicenter phase II trial. J Clin Oncol 1997;15:3185–3191.
6. Alberts DS, García DJ. Safety aspects of pegylated liposomal doxorubicin in patients with cancer. Drugs 1997; 54(Suppl 4):30–35.
7. Berry G, Billingham M, Alderman E, et al. The use of cardiac biopsy to demonstrate reduced cardiotoxicity in AIDS Kaposi's sarcoma patients treated with pegylated liposomal doxorubicin. Ann Oncol 1998;9:711–716.
8. Muggia FM, Hainsworth JD, Jeffers S, et al. Phase II study of liposomal doxorubicin in refractory ovarian cancer: Antitumor activity and toxicity modification by liposomal encapsulation. J Clin Oncol 1997;15:987–993.
9. Miller AB, Hoogstraten B, Staquet M. Reporting results of cancer treatment. Cancer 1981;47:207–214.

10. Aaronson NK, Ahmedzai S, Bergman B, et al. The European Organization for Research and Treatment of Cancer QLQ-C30: A quality-of-life instrument for use in international trials in oncology. J Natl Cancer Inst 1993;85: 363–376.
11. Fayers PM, Aaronson NK, Bjordal K, et al. The EORTC QLQ-C30 Scoring Manual. 3rd ed. Brussels: European Organization for Research and Treatment of Cancer. 2001.
12. Simon R. Optimal two-stage designs for phase II trials. Control Clin Trials 1989;10:1–10.
13. Cronbach LJ. Coefficient alfa and the internal structure of test. Psychometrika 1951:16;297–334.
14. Fitzpatrick JE. The cutaneous histopathology of chemotherapeutic reactions. J Cutan Pathol 1993;20:1–14.
15. Toma S, Tuccie A, Villani G, et al. Liposomal doxorubicin (Caelyx) in advanced pretreated soft tissue sarcomas: A phase II study of the Italian Sarcoma Group (ISG). Anticancer Res 2000;20:485–492.
16. Skubitz KM. Phase II trial of pegylated-liposomal doxorubicin (Doxil™) in sarcoma. Cancer Invest 2003;21:167–176.
17. García AA, Kempf RA, Rogers M, et al. A phase II study of Doxil (liposomal doxorubicin): Lack of activity in poor prognosis soft tissue sarcomas. Ann Oncol 1998;9: 1131–1133.
18. Chidiac T, Budd GT, Pelley F, et al. Phase II trial of liposomal doxorubicin (Doxil®) in advanced soft tissue sarcomas. Invest New Drugs 2000;18:253–259.
19. Judson I, Radford JA, Harris M, et al. Randomised phase II trial of pegylated liposomal doxorubicin (DOXIL®/ CAELYX®) versus doxorubicin in the treatment of advanced or metastatic soft tissue sarcoma: A study by the EORTC Soft Tissue and Bone Sarcoma Group. Eur J Cancer 2001;37:870–877.
20. van Glabbeke M, Verweij J, Judson I, et al. Progression-free rate as the principal end-point for phase II trials in soft-tissue sarcomas. Eur J Cancer 2002;38:543–549.
21. Gordon AN, Granai CO, Rose PG, et al. Phase II study of liposomal doxorubicin in platinum- and paclitaxel-refractory epithelial ovarian cancer. J Clin Oncol 2000;18:3093–3100.
22. Lyass O, Uziely B, Ben-Yosef R, et al. Correlation of toxicity with pharmacokinetics of pegylated liposomal doxorubicin (Doxil®) in metastatic breast carcinoma. Cancer 2000;89:1037–47.
23. Hamilton A, Biganzoli L, Coleman R, et al. EORTC 10968: A phase I clinical and pharmacokinetic study of polyethylene glycol liposomal doxorubicin (Caelyx®, Doxil®) at a 6-week interval in patients with metastatic breast cancer. Ann Oncol 2002;13:910–918.
24. O'Brien MER, Wigler N, Inbar M, et al. Reduced cardiotoxicity and comparable efficacy in a phase III trial of pegylated liposomal doxorubicin HCl (CAELYX™/ Doxil®) versus conventional doxorubicin for first-line treatment of metastatic breast cancer. Ann Oncol 2004;15: 440–449.

